# The landscape of antibiotic usage among COVID-19 patients in the early phase of pandemic: a Malaysian national perspective

**DOI:** 10.1186/s40545-022-00404-4

**Published:** 2022-01-11

**Authors:** Izzati-Nadhirah Mohamad, Calvin Ke-Wen Wong, Chii-Chii Chew, E-Li Leong, Biing-Horng Lee, Cheng-Keat Moh, Komalah Chenasammy, Steven Chee-Loon Lim, Hong-Bee Ker

**Affiliations:** 1 Medical Department, Hospital Raja Permaisuri Bainun, Ministry of Health, Ipoh, Malaysia; 2 Clinical Research Centre, Hospital Raja Permaisuri Bainun, Ministry of Health, Ipoh, Malaysia; 3 Infectious Diseases Unit, Medical Department, Hospital Raja Permaisuri Bainun, Ministry of Health, Ipoh, Malaysia

**Keywords:** Anti-bacterial agents, COVID-19, Prevalence, Infectious disease, Malaysia, Antibiotic use

## Abstract

**Background:**

During the early phase of the COVID-19 pandemic, antibiotic usage among COVID-19 patients was noted to be high in many countries. The objective of this study was to determine the prevalence of antibiotic usage and factors affecting antibiotic usage among COVID-19 patients during the early phase of the COVID-19 pandemic in Malaysia.

**Methods:**

This was a cross-sectional study that involved reviewing medical records of COVID-19 Malaysian patients aged 12 and above who were diagnosed with COVID-19 and received treatment in 18 COVID-19 hospitals from February to April 2020. A minimum sample of 375 patients was required. A binary logistic regression analysis was performed to determine factors associated with antibiotic usage. Variables with *p* < 0.05 were considered statistically significant.

**Results:**

A total of 4043 cases were included for analysis. The majority of the patients (87.6%) were non-smokers, male (65.0%), and had at least one comorbidity (37.0%). The median age was 35 years (IQR: 38). The prevalence of antibiotic usage was 17.1%, with 5.5% of them being prescribed with two or more types of antibiotics. The most frequent antibiotics prescribed were amoxicillin/clavulanic acid (37.8%), ceftriaxone (12.3%), piperacillin/tazobactam (13.3%), azithromycin (8.3%), and meropenem (7.0%). Male patients (adjusted OR 1.53), who had a comorbidity (adjusted OR 1.36), associated with more severe stage of COVID-19 (adjusted OR 6.50–37.06), out-of-normal range inflammatory blood parameters for neutrophils, lymphocytes, and C-reactive protein (adjusted OR 2.04–3.93), corticosteroid use (adjusted OR 3.05), and ICU/HDU admission (adjusted OR 2.73) had higher odds of antibiotic use.

**Conclusions:**

The prevalence of antibiotic usage in the early phase of the COVID-19 pandemic was low, with amoxicillin/clavulanic acid as the most common antibiotic of choice. The study showed that clinicians rationalized antibiotic usage based on clinical assessment, supported by relevant laboratory parameters.

## Background

In the early phase of the COVID-19 pandemic, the virus infected more than 3 million people, causing about 200,000 deaths globally as of April 30, 2020 [1]. In this period, the prevalence of SAR-CoV-2 co-infection is low (14 to 25%), with bacterial co-infection ranging from 5 to 11% [2]. Despite a relatively low bacterial co-infection rate, the prevalence of antibiotic usage in patients with COVID-19 infection was nevertheless considerably high. A research rapidly reviews studies researching on antibiotic usage in the early phase of pandemic revealed that 82.3% of 10, 329 COVID-19 patients (82.3%) received antibiotics therapy without regard to the severity of COVID-19 infection [3]. A meta-analysis shows that approximately 75% of patients with COVID-19 admitted to hospitals were given an antibiotic; yet, only 8% of the patients were confirmed with bacterial co-infection [4]. A similar pattern is seen among critically ill patients in which the rate of superinfection in COVID-19 patients was 13.5%, but 75.4 to 94% of them were treated with antibiotics [3, 5].

In the early outbreak of COVID-19, the nature of the disease was not clearly understood and lacked guidelines in managing COVID-19 patients with bacterial co-infection. Most of the physicians (83%) from 23 countries reported adapting local community-acquired pneumonia for antibiotic use in patients with COVID-19 with bacterial co-infection [6]. The antibiotic usage was shown to be biphasic in a study from the West, with amoxicillin/clavulanate being the most commonly prescribed antibiotic during the first wave of the pandemic for empirical coverage of all COVID-19 pneumonia cases, while other broad-spectrum antibiotics being the most commonly prescribed antibiotic during the second wave in April 2020, with a slight decrease in amoxicillin/clavulanate usage when more patients were admitted to intensive care units (ICU) [7]. This coincides with a study that showed that there was a peak of new ICU admissions in April 2020 [8]. When patients progress into severe disease and require critical care, broad-spectrum antibiotics are used empirically or prophylactically to prevent secondary bacterial infection [9]. The top usage of antibiotics in COVID-19 patients suspected of bacterial co-infection is azithromycin, ceftriaxone, cefepime, moxifloxacin, meropenem, and piperacillin/tazobactam [3, 10]. Consistently, physicians reported that β-lactams plus β-lactamase inhibitors or macrolides or fluoroquinolones alone (52.4%) were most frequently prescribed to COVID-19 patients at the beginning of the pandemic outbreak. In patients who were critically ill, piperacillin/tazobactam was the most commonly prescribed antibiotic [11].

Independent factors that affect the decision of a physician in prescribing an antibiotic include advanced age, presence of comorbidity, February–March 2020 admissions, symptoms of dry cough, fever, dyspnoea, flu-like, and elevated C-reactive protein (CRP) biomarkers [4, 12], procalcitonin [11], long hospital length of stay with a median of 12 days, admission to the ICU, and the necessity for mechanical ventilation [13]. Given variations in the management of COVID-19 in different countries, where some countries admit all COVID-19 patients while others only admit patients with severe symptoms, the factors affecting the use of antibiotic agents could be varied and influenced by the health policy that is unique to each country.

The distribution of antibiotic usage according to different region in the world, despite varying, it is considerably substantial. East and Southeast Asia (excluding China) reporting the highest (87.5%) and Europe the lowest (63.1%) [4]. Malaysia being one of the Southeast Asian countries, we are uncertain if the usage of antibiotics is as high as those reported in other countries in the same region. Additionally, reporting this data on a national scale would allow researchers and clinicians to gain insight into the extent of antibiotic usage in comparison to other countries, usage before the pandemic and the latter phase of the pandemic. This would also assist clinicians to estimate if multidrug-resistant bacteria is likely to occur in the future if there is any overuse of antibiotic agents [14]. This study aimed to determine the prevalence of antibiotic usage, the types of antibiotic agents, and the associated risk factors of COVID-19 patients who received antibiotics from a national perspective during the early phase of the COVID-19 pandemic.

## Methods

### Study design

This retrospective study involved reviewing medical records of COVID-19 positive patients. All Malaysian patients aged 12 years and above, who were RT-PCR confirmed COVID-19 and received treatment in 18 government-funded COVID-19 hospitals from 1st of February to 30th of April in 2020 were sampled. Medical officers who treated these patients were in-charge in entering the patients’ medical records real time into a national database (REDCap© system) developed and managed by the Infectious Diseases Unit and Clinical Research Centre of Hospital Sungai Buloh.

Approval was granted for researchers to access data relevant to antibiotic use. The administrator of the database helped to extract anonymous patients’ details including social demography (age, gender, smoking status, comorbidity), symptoms when presenting to the healthcare facilities, duration of hospitalization, types of ward admitted (normal ward, ICU or high dependency unit [HDU]), clinical severity stage [15], antibiotic regime, corticosteroid usage, full blood count (FBC) and renal function tests on admission, CRP, requirement of oxygen, and patient outcome (alive at the point of discharge or transfer to secondary healthcare facilities for continued observation or death). While there was lack of clear guidance for handling microbiological specimens early in the pandemic, cultures and sensitivity test was not conducted on COVID-19 patient suspected with bacterial co-infection [16]. Hence, the microbiology data were not available. Of note, stages 1 to 3 for clinical severity for COVID-19 classification use in Malaysia [15] correspond to non-severe, while stages 4 and 5, respectively, correspond to severe and critical categories defined by the World Health Organization for COVID-19 severity [14] (Table 1).

**Table 1:** The stages of COVID-19 severity in Malaysia corresponding to the classification of World Health Organization

Clinical stage of COVID-19 severity in Malaysia [15]		COVID-19 severity based on World Health Organization [14]	
Stage 1	Asymptomatic	Non-severe	Absence of sign of severe or critical disease
Stage 2	Symptomatic, no pneumonia		
Stage 3	Symptomatic, pneumonia		
Stage 4	Symptomatic, pneumonia, requiring supplemental oxygen	Severe	• SpO_2_ < 90% on room air • Respiratory rate > 30 breaths/min in adults • Raised respiratory rate in children • Signs of severe respiratory distress
Stage 5	Critically ill with multi-organ involvement	Critical	• Acute respiratory distress syndrome • Sepsis • Septic shock • Require life- sustaining therapies

*SpO*_*2*_ oxygen saturation via pulse oximeter

### Sample size and sampling method

The prevalence of antibiotic usage among patients diagnosed with COVID-19 during early phase of pandemic reported by other countries ranged from 58 to 71% [11]. By using sample size calculation for prevalence study [17], a minimum sample size of 375 was required to achieve precision of 5% and confidence level of 95% when 58% of the patients with COVID-19 were assumed to be prescribed with an antibiotic upon admission to the hospital. To gain an overview of antibiotic usage in the national perspectives, all medical records of the COVID-19 patients that fulfilled the inclusion were sampled.

### Data analysis

IBM SPSS® Statistics Version 20.0 was used to perform descriptive analysis on the data. The prevalence of antibiotic usage was calculated as the proportion of patients prescribed at least one antibiotic. Antibiotics were further classified according to the antibiotic drug class. A binary logistic regression analysis was performed to determine factors associated with the prescribing of antibiotics during hospitalization. Univariate analysis was employed to determine the variables with a *p*-value < 0.05 that were needed for multivariate analysis. The variables included for multivariate analysis include age, gender, number of comorbidities, smoking status, clinical severity on admission, the most severe clinical classification during hospitalization, white blood cell count, platelets, neutrophils, lymphocytes, CRP, duration of hospitalization, use of anti-viral agents, use of anti-malarial agents, corticosteroid therapy and ICU/HDU admission. Stepwise backward logistic regression analysis was used to determine the final model that predicts the use of antibiotics when a COVID-19 patient is hospitalized. There was no detectable multicollinearity between the variables. Model fitness was tested using the Hosmer–Lemeshow goodness-of-fit test.

## Results

The medical records of 4043 COVID-19 patients were included for analysis after eliminating 840 cases consisting of foreigners and children under the age of 12. Their age ranged between 12 and 95 years, with the median age of 38 (IQR: 38.0). Most of them were male (65.0%), Malay (77.2%), had hypertension (20.4%), and were non-smokers (87.6%). One-fifth (20.6%) had one comorbidity, while approximately 16% had at least two comorbidities, and a patient with seven comorbidities was noted (Table 2).

**Table 2:** Demographic characteristic of COVID-19 patients, (*n* = 4043)

Variables		*n* (%)	Antibiotic use	*p*-value*
Age	≤ 65 years	3720 (92.0)	572 (15.4)	< 0.001
	> 65 years, median (IQR)	323 (8.0)	126 (38.9)	
Gender	Male	2628 (65.0)	488 (18.6)	< 0.001
	Female	1415 (35.0)	21 (14.8)	
Ethnicity	Malay	3119 (77.2)	498 (16.0)	< 0.001^a^
	Chinese	333 (8.2)	67 (20.1)	
	Indian	115 (2.8)	18 (15.7)	
	Orang Asli Semenanjung	4 (0.1)	0 (0.0)	
	Bumiputera Sabah	191 (4.7)	64 (33.5)	
	Bumiputera Sarawak	281 (7.0)	51 (18.1)	
Number of comorbidities	No comorbidity	2549	258 (10.1)	< 0.001^b^
	1	834 (20.6)	205 (24.6)	
	2	373 (9.2)	120 (32.2)	
	3	194 (4.8)	74 (38.1)	
	4	68 (1.7)	31 (45.6)	
	5	20 (0.5)	7 (35.0)	
	6	4 (0.1)	2 (50.0)	
	7	1 (0.02)	1 (100.0)	
Types of comorbidities	Hypertension	824 (20.4)	270 (32.8)	< 0.001
	Diabetes mellitus	517 (12.8)	204 (39.5)	< 0.001
	Dyslipidaemia	215 (5.3)	49 (22.8)	0.028
	Asthma	176 (4.4)	35 (19.9)	0.347
	Chronic cardiac disease	172 (4.3)	64 (37.2)	< 0.001
	Chronic kidney disease	92 (2.3)	60 (65.2)	< 0.001
	Obesity	73 (1.8)	19 (26.0)	0.046
	Malignant neoplasm	40 (1.0)	15 (37.5)	0.001
	Rheumatologic disorder	33 (0.8)	13 (39.4)	0.001
	Chronic neurological disorder	32 (0.8)	12 (37.5)	0.002
	Chronic pulmonary disease	28 (0.7)	19 (67.9)	< 0.001
	Liver disease	10 (0.3)	2 (20.0)	0.822
	Chronic hematologic disease	7 (0.2)	0 (0.0)	0.103^#^
	AIDS/HIV	7 (0.2)	0 (0.0)	0.103
	Dementia	4 (0.1)	1 (25.0)	0.697^#^
Smoking	Smoker	500 (12.4)	53 (16.9)	0.851
	Non-smoker	3543 (87.6)	645 (17.3)	

*IQR* interquartile range; *AIDS* acquired immunodeficiency syndrome; *HIV* human immunodeficiency virus

^a^ Chi-square analysis of Malay and non-Malay ethnicity

^b^ Chi-square analysis of no comorbidity vs 1, 2 and more than 2 comorbidities

* Chi-Square test

^#^ Fisher's Exact test

Most of them presented with symptoms of fever (38.7%) and cough (40.1%). They were classified stage 1 (40.4%) and stage 2 (36.8%) upon admission to the hospitals. The majority of them remained in stage 1 (33.3%) and stage 2 (33.3%). The proportion of patients at stage 3 increased from 15.9 to 22.2% during the hospital stay. Notably, the number of patients at stage 5 was increased from 57 (1.4%) on admission to 169 (4.2%) during hospitalization. Most of the biomarkers were normal for FBC and renal function tests except haematocrit (68.3%) and serum creatinine (58.0%). About one-tenth of them had abnormal profile in platelet (11.4%) and neutrophil counts (11.6%). The median duration of hospitalization was 11 days (IQR: 9.00). Five percent (*n* = 193) required intensive care, while 2% (*n* = 77) died (Table 3).

**Table 3:** Clinical characteristic of COVID-19 patients, (*n* = 4043)

Variables		*n* (%)	Antibiotic use^^^	*p-*value*
Symptoms of presentation	Cough	1620 (40.1)	457 (28.2)	< 0.001
	Fever	1565 (38.7)	433 (27.7)	< 0.001
	Sore throat	713 (17.6)	149 (20.9)	0.005
	Rhinorrhoea	550 (13.6)	104 (18.9)	0.272
	Dyspnoea	284 (7.0)	142 (50.0)	< 0.001
	Diarrhoea	279 (6.9)	100 (35.8)	< 0.001
	Myalgia	217 (5.4)	52 (24.0)	0.007
	Malaise	183 (4.5)	72 (38.7)	< 0.001
	Anosmia	155 (3.8)	10 (6.5)	< 0.001
	Headache	154 (3.8)	26 (16.9)	0.898
	Arthralgia	114 (2.8)	29 (25.4)	0.019
	Nausea/vomiting	100 (2.5)	38 (38.0)	< 0.001
	Ageusia	38 (0.9)	3 (7.9)	0.125^#^
	Abdominal pain	36 (0.9)	13 (36.1)	0.003
	Chest pain	50 (1.2)	21 (42.0)	< 0.001
	Skin rash	9 (0.2)	2 (22.2)	0.659^#^
	Conjunctivitis	9 (0.2)	0 (0.0)	0.374^#^
	Wheezing	6 (0.1)	3 (50.0)	0.104^#^
	Ear pain	5 (0.1)	0 (0.0)	0.168^#^
	Haemorrhage	3 (0.1)	3 (100.0)	< 0.005^#^
Clinical severity stage on admission^#^	Stage 1	1634 (40.4)	64 (3.9)	< 0.001
	Stage 2	1487 (36.8)	244 (16.4)	
	Stage 3	644 (15.9)	186 (28.9)	
	Stage 4	221 (5.5)	145 (69.0)	
	Stage 5	57 (1.4)	51 (17.1)	
Worst clinical stage^#^	Stage 1	1345 (33.3)	16 (1.2)	< 0.001
	Stage 2	1347 (33.3)	154 (11.4)	
	Stage 3	896 (22.2)	200 (22.3)	
	Stage 4	287 (7.1)	166 (60.1)	
	Stage 5	168 (4.2)	157 (93.5)	
Out of normal range FBC (on admission)	White blood cells (*n* = 3661)	302 (8.3)	111 (15.9)	< 0.001
	Haemoglobin (*n* = 3661)	146 (4.0)	65 (9.3)	< 0.001
	Hematocrit (*n* = 3638)	2486 (68.3)	271 (39.3)	< 0.001
	Platelets (*n* = 3661)	417 (11.4)	102 (14.6)	< 0.001
	Neutrophil count (*n* = 3548)	413 (11.6)	152 (21.8)	< 0.001
	Lymphocyte count (*n* = 3573)	141 (4.0)	55 (7.9)	< 0.001
Out of normal range renal function test (on admission)	Serum creatinine (*n* = 3493)	2025 (58.0)	367 (52.6)	0.005
	Blood urea nitrogen (*n* = 3505)	678 (19.3)	228 (32.7)	< 0.001
	Sodium (*n* = 3507)	469 (13.4)	230 (33.0)	< 0.001
	Potassium (*n* = 3500)	855 (24.4)	186 (26.6)	< 0.001
Out of normal range CRP (*n* = 2522)		346 (13.7)	188 (26.9)	< 0.001
Use of anti-fungal agents		50 (1.2)	40 (80.0)	< 0.001
Use of anti-malarial agents		1935 (47.9)	570 (29.5)	< 0.001
Use of anti-viral agents		1020 (33.6)	549 (53.8)	< 0.001
Oxygen therapy		459 (11.4)	329 (71.7)	< 0.001
Corticosteroid therapy		124 (3.1)	108 (87.1)	< 0.001
Admission to ICU/HDU		192 (4.8)	176 (91.7)	< 0.001
Duration of hospitalization*, median (IQR)*		11.00 (9.00)		< 0.001^§^
Patient outcome	Alive	3966 (98.1)	631 (15.9)	< 0.001
	Death	77 (1.9)	67 (87.0)	

^#^1: asymptomatic, 2: symptomatic, no pneumonia, 3: symptomatic, pneumonia, 4: symptomatic, pneumonia, requiring supplemental oxygen, 5: critically ill with multi-organ involvement

*IQR* interquartile range; *FBC* full blood counts; *CRP* C-reactive protein; *ICU* Intensive Care Unit; *HDU* High Dependency Unit

^^^ Antibiotic use during hospitalization

^#^ Fisher's Exact test

* Chi-Square test

^§^ Mann–Whitney *U* test

The number of patients prescribed antibiotic treatment increased from 524 (13.0%) on admission to 690 (17.1%) patients during hospitalization. Of those who were prescribed antibiotics, most of them were given one antibiotic (67.7–82.1%) and about 13 to 16% of them received two antibiotic therapies. Notably, two patients were given nine antibiotics throughout hospital stay. Patients who were administered at least three types of antibiotics increased from 25 (4.8%) to 107 (15.5%) during the period of admission to hospitalization.

The increased usage of antibiotics from admission to hospitalization was seen in β-lactam plus β-lactamase inhibitor, third and fourth generation cephalosporin, macrolides, penicillin, carbapenems, vancomycin, polymyxins, fluoroquinolones, and co-trimoxazole. The most commonly prescribed antibiotics upon patient admission were amoxicillin plus clavulanic acid, ceftriaxone and azithromycin. Meanwhile, addition of piperacillin/tazobactam, and meropenem were the most frequently given antibiotics during hospitalization (Table 4).

**Table 4:** Antibiotics and related treatments for COVID-19 patients during admission and hospitalization

Variable		Admission, *n* (%) *n* = 524^a^	Hospitalization, *n* (%) *n* = 690^a^
Number of antibiotic prescribed per patient	1	430 (82.1)	467 (67.7)
	2	69 (13.2)	116 (16.8)
	3	16 (3.1)	52 (7.5)
	4	5 (1.0)	24 (3.5)
	5	2 (0.4)	15 (2.3)
	6	0 (0.0)	11 (1.6)
	7	1 (0.2)	1 (0.1)
	8	1 (0.2)	2 (0.3)
	9	0 (0.0)	2 (0.3)
Fluoroquinolones	Ciprofloxacin	1 (0.2)	4 (0.4)
	Moxifloxacin	1 (0.2)	1 (0.1)
Cephalosporins	Fourth generation: cefepime	12 (1.8)	49 (4.3)
	Third generation: ceftriaxone	81 (12.3)	139 (12.3)
	Third generation: cefoperazone	1 (0.2)	0 (0.0)
	Third generation: ceftazidime	6 (0.9)	16 (1.4)
	Second generation: cefuroxime	5 (0.8)	11 (1.0)
	First generation: cephalexin	1 (0.2)	2 (0.2)
	First generation: cefazolin	1 (0.2)	2 (0.2)
Macrolides	Erythromycin	3 (0.5)	3 (0.3)
	Azithromycin	64 (9.7)	94 (8.3)
Glycopeptides	Vancomycin	4 (0.6)	20 (1.8)
Polymyxins	Colistin	2 (0.3)	19 (1.7)
	Polymyxin B	1 (0.2)	10 (0.9)
Β-lactam/β-lactamase inhibitor	Amoxicillin/clavulanic acid	375 (56.7)	428 (37.8)
	Piperacillin/tazobactam	53 (8.0)	151 (13.3)
	Ampicillin/sulbactam	16 (2.4)	43 (3.8)
Penicillin	Amoxicillin	3 (0.5)	6 (0.5)
	Cloxacillin	7 (1.1)	19 (1.7)
Tetracycline	Doxycycline	2 (0.3)	9 (0.8)
Lincosamides	Clindamycin	2 (0.3)	4 (0.4)
Co-trimoxazole		4 (0.6)	16 (1.4)
Carbapenem	Imipenem	0 (0.0)	4 (0.4)
	Meropenem	12 (1.8)	79 (7.0)
Nitroimidazoles	Metronidazole	4 (0.6)	4 (0.4)
Corticosteroid usage (*n* = 4043)		–	124 (3.1)
Oxygen therapy (*n* = 4043)	Non-invasive	221 (5.4)	324 (8.0)
	Invasive	50 (1.2)	135 (3.3)

^a^Total number of patients prescribed antibiotic(s)

A higher odds of antibiotics usage was observed among patients who were male (adjusted OR [AOR] 1.53), had a comorbidity (AOR 1.36), with severity of disease classification of stage 2 to 5 (AOR 4.32 to 30.85), tested out-of-normal range biomarkers for neutrophil (AOR 2.31), lymphocyte counts (AOR 2.04), and CRP (AOR 3.34), hospitalized more than 7 days (AOR 1.89 to 3.81), had corticosteroids therapy (AOR 3.05), prescribed with anti-viral agents (AOR 6.38) and anti-malarial agents (AOR 1.63), and had admitted to HDU or ICU (AOR 2.73) (Table 5).

**Table 5:** Factors associated with antibiotics usage among COVID-19 during hospitalization

Variables		Simple logistic regression				Multiple logistic regression			
		Crude *OR*	95% CI	*χ*^2^ statistics (*df*)^a^	*p*-value^a^	Adj. *OR*	95% CI	*χ*^2^ statistics (*df*)^a^	*p*-value
Age (years)	≤ 65	1.00							
	> 65	3.56	2.78, 4.53	96.42 (1)	< 0.001	–	–	–	–
Gender	Female	1.00				1.00			
	Male	1.35	1.13, 1.61	11.03 (1)	0.001	1.53	1.20, 1.96	11.91 (1)	0.001
Smoking Status	Non-smoker	1.00							
	Smoker	1.16	0.91, 1.48	1.47 (1)	0.225	–	–	–	–
Comorbidity				260.9 (2)	< 0.001			4.65 (2)	0.098
	No comorbidity	1.00				1.00			
	1 comorbidity	2.94	2.40, 3.61	106.8 (1)^b^	< 0.001^b^	1.36	1.03, 1.79	4.91 (1)^b^	0.031^b^
	≥ 2 comorbidities	4.95	4.03, 6.08	232.2 (1)^b^	< 0.001^b^	1.09	0.80, 1.49	0.41 (1)^b^	0.576 ^b^
Worst case classification				1179.8 (4)	< 0.001			98.45 (4)	< 0.001
	Stage 1	1.00				1.00			
	Stage 2	11.91	6.85, 20.72	77.04 (1)^b^	< 0.001^b^	6.50	3.61, 11.69	38.93 (1)^b^	< 0.001^b^
	Stage 3	27.14	15.67, 47.03	138.6 (1)^b^	< 0.001^b^	4.07	2.11, 7.84	17.64 (1)^b^	< 0.001^b^
	Stage 4	138.14	77.59, 245.9	280.4 (1)^b^	< 0.001^b^	10.38	5.08, 21.19	41.27 (1)^b^	< 0.001^b^
	Stage 5	1356.93	605.6, 3040.6	307.0 (1)^b^	< 0.001^b^	37.06	13.04, 105.3	44.96 (1)^b^	< 0.001^b^
White blood cell				80.70 (2)	< 0.001			25.35 (2)	< 0.001
	Normal range	1.00							
	Out of normal range	3.05	2.38, 3.93	75.72 (1)	< 0.001^b^	1.23	0.76, 1.97	0.35 (1)^b^	0.555^b^
	Not tested	0.65	0.47, 0.91	6.45 (1)	0.011^b^	0.19	0.09, 0.37	21.93 (1)^b^	< 0.001^b^
Platelets				24.91 (2)	< 0.001	–	–	–	–
	Normal range	1.00							
	Out of normal range	1.58	1.24, 2.01	13.55 (1)	< 0.001^b^				
	Not tested	0.61	0.44, 0.85	8.49 (1)	0.004^b^				
Neutrophils				109.0 (2)	< 0.001			14.81 (2)	0.001
	Normal range	1.00				1.00			
	Out of normal range	3.48	2.78, 4.35	119.3 (1)	< 0.001^b^	2.31	1.51, 3.53	15.34 (1)^b^	< 0.001^b^
	Not tested	1.29	1.00, 1.66	3.95 (1)	0.047^b^	1.18	0.34, 4.33	0.06 (1)^b^	0.803^b^
Lymphocytes				40.81 (2)	< 0.001			10.88 (2)	0.004
	Normal range	1.00				1.00			
	Out of normal range	3.34	2.33, 4.69	44.63 (1)	< 0.001^b^	2.04	1.18, 3.51	6.47 (1)^b^	0.011^b^
	Not tested	1.14	0.88, 1.47	7.01 (1)	0.317^b^	4.21	1.08, 16.38	3.53 (1)^b^	0.060^b^
C-reactive protein				439.9 (2)	< 0.001			57.26 (2)	< 0.001
	Normal range	1.00				1.00			
	Out of normal range	14.49	11.12, 18.87	392.5 (1)	< 0.001^b^	3.34	2.33, 4.78	48.38 (1)^b^	< 0.001^b^
	Not tested	3.46	2.83, 4.22	148.6 (1)	< 0.001^b^	2.32	1.75, 3.08	45.69 (1)^b^	< 0.001^b^
Duration of hospitalization				246.2 (3)	< 0.001			52.91 (1)	< 0.001
	7 days or less	1.00				1.00			
	8 to 11 days	1.62	1.20, 2.19	9.97	0.002	1.89	1.26, 2.86	9.25 (1)	0.002
	12- 16 days	3.01	2.26, 4.01	56.47	< 0.001	2.59	1.74, 3.86	21.76 (1)	< 0.001
	17 days or more	6.10	4.64, 8.03	167.1	< 0.001	3.81	2.57, 5.67	44.16 (1)	< 0.001
Use of anti-viral agents	Not administered	1.00							
	Administered	22.77	18.51, 28.02	1121.9 (1)	< 0.001	6.38	4.68, 8.70	150.5 (1)	< 0.001
Use of anti-malarial agents	Not administered	1.00							
	Administered	6.46	5.27, 7.92	408.78 (1)	< 0.001	1.63	1.19, 2.25	9.35 (1)	0.002
Use of anti-fungal agents	Not administered	1.00							
	Administered	20.27	10.09, 40.74	96.02 (1)	< 0.001	–	–	–	–
Corticosteroid therapy	Not administered	1.00				1.00			
	Administered	38.70	22.73, 65.91	306.6 (1)	< 0.001	3.05	1.48, 6.25	10.15 (1)	0.001
ICU/HDU admission	No	1.00				1.00			
	Yes	71.41	42.45, 120.1	558.3 (1)	< 0.001	2.73	1.34, 5.58	8.05 (1)	0.005
Oxygen therapy	No	0.00							
	Yes	22.29	17.71, 28.05	799.9 (1)	< 0.001	–	–	–	–

*OR* odds ratio, *CI* confidence interval, *ICU/HDU* Intensive Care Unit/High Dependency Unit

^a^Likelihood ratio test

^b^Wald test

## Discussion

This study assessed the use of antibiotics in the early wave of the COVID-19 outbreak between February and April 2020 in Malaysia. At the beginning of the pandemic, all patients confirmed positive for COVID-19 infection were required to be admitted to government-funded facilities [18]. Therefore, we were able to include all the medical records of Malaysian patients with COVID-19 in the early phase of the pandemic. It allowed us to conduct a thorough analysis of the prevalence of antibiotic use and the choice of antibiotic agent prescribed to the COVID-19 patients suspected of bacterial co-infection. The factors associated with antibiotic use were critical information that allowed clinicians to make decisions when prescribing antibiotics for the COVID-19 suspected bacterial co-infection, especially when microbiological testing was not permitted in certain conditions. The study findings provide a full picture of antibiotic use during the early phase of pandemic and permit researchers to conduct further research on the changes in the antibiotic usage pattern at different phases of COVID-19.

Our study showed that the national antibiotic usage in this country was below 20% in the early outbreak of COVID-19. In the early phase of the pandemic, 67% of COVID-19 patients were treated with empirical antibiotics in the United States, [10]. A higher percentage of antibiotic usage was seen in China and Indonesia (70.5 to 95.0%) [19–22]. The high prevalence of antibiotics usage in the pandemic was further confirmed by two meta-analyses [23, 24], in which 71.9 to 74.0% of COVID-19 patients were given antibiotics, where only 1.0 to 19.7% were confirmed with bacterial co-infection [10, 20, 21, 23–27]. The prevalence of national antibiotic usage among COVID-19 patients in Malaysia was relatively lower compared to that of other countries. This can be attributed to the initial understanding among the Infectious Disease Team in Malaysia during the early phase of pandemic that COVID-19 is a viral infection and is in line with the recommendation of the World Health Organization where antibiotics should not be considered unless suspicious of bacterial co-infection in COVID-19 patients [16, 28]. Hence, the antibiotic treatment regime was not included in the Malaysian guidelines for clinical management of COVID-19 confirmed cases [16]. The antibiotic treatment guide was added to the guideline during the later stages of the pandemic, stating that where there is evidence of bacterial co-infections, antibiotics should be given without waiting for microbiological results [29].

The types of antibiotics used in this study were similar to those used in a study looking into antibiotic usage in a few Western countries [11], which preferred β-lactam/β-lactamase inhibitors, cephalosporins, and macrolides. If a patient was in ICU or required oxygen therapy, piperacillin/tazobactam and meropenem were the choices. Despite the fact that information on antibiotic regimes was not included in the interim Malaysian guidelines for clinical management of COVID-19 [16], the choices of antibiotic usage for COVID-19 patients with suspected bacterial co-infection were as recommended in the Malaysian National Antibiotic Guideline 2019 for pneumonia [30]. In Spain, amoxicillin and amoxicillin/clavulanic acid were the favourites (72.0%) [31], but they were prescribed at a higher rate in comparison with our study (37.8 to 56.7%). Although ceftriaxone was the second most common antibiotic used among COVID-19 patients in other countries such as the United States and Bangladesh, where it was prescribed at a rate of nearly 54.0% [10, 32], the usage was low in this study (12.3%). In a meta-analysis, fluoroquinolone and 3rd generation cephalosporins accounted for 56.8 to 76.0% of the total antibiotic prescriptions [4, 21, 23, 33], which was substantially higher than the local usage of 0.2% to 12.3%. With regard to the carbapenem group of antibiotics, developing countries like Bangladesh prescribed 40.9% as compared to this country, which recorded a single-digit percentage of about 7% carbapenem utility during hospitalization [32]. The difference may be attributed to the different healthcare systems, the provision of antibiotics and infection control measures taken during the pandemic.

Notably, anti-malarial and anti-viral agents were prescribed to nearly half and one-third of the COVID-19 patients, respectively. This was attributed to the interim Malaysian guidelines for clinical management of COVID-19 patients, which recommend that those with disease severity of stage 2 to 5, which is equivalent to the World Health Organization's mild to critical severity classification [28], be given hydroxychloroquine 400 mg BD for one day and followed by 200 mg twice daily (BID), and the addition of lopinavir/ritonavir 2 BID, and ribavirin 2.4 g immediately and 1.2 g BID if needed after consulting with an infectious disease physician [16]. Initial evidence suggested that the anti-viral properties of hydroxychloroquine were associated with a significant reduction of viral load and that this effect could be enhanced by combining with azithromycin [34]. Nevertheless, a combination of azithromycin and hydroxychloroquine was not included in the recommendation of the interim Malaysian guidelines for clinical management of COVID-19, and hence the usage of azithromycin was low (8.3 to 9.7%). Nonetheless, in the latter stages of the pandemic, the treatment regimen was phased out of the COVID-19 clinical management guidance due to a lack of quality evidence proving its efficacy and associated with severe side effects such as QT prolongation or cardiotoxicity [14, 28, 29].

The patients who were more ill tended to get antibiotic coverage as their inflammatory markers were elevated, they had a longer duration of hospitalization, were given anti-viral and anti-malarial agents, were treated in the ICU and required corticosteroids. These factors are consistent with regard to the interim Malaysia Ministry of Health guidelines for patients who are critically ill. The recommendation was to consider starting on antibiotics [35]. Chedid et al*.* showed in the meta-analysis that the higher the severity in terms of clinical severity, the higher the number of patients who were given antibiotics (severe 100% vs. moderate 67.9%) [23]. Elevated inflammatory markers such as CRP and procalcitonin, together with leukocytosis and increased neutrophil count, were associated with higher antibiotic prescriptions [21, 36]. This could be due to the cytokine release syndrome that produces signs and symptoms that may be difficult to distinguish from sepsis or septic shock due to bacterial infections. Suleyman et al. and Seaton et al. found that comorbidities were also a factor taken into consideration by clinicians before starting antibiotics [37, 38].

### Limitations

Some inevitable limitations associated with the extraction of data from a large national database include missing data and possibly some inaccurate information entered. Nevertheless, the clinical records of COVID-19 were entered into the database in real time by the medical officer in-charge, and recall bias was avoided. The information regarding antibiotic dosage, duration of therapy, and imaging studies was incomplete and, thus, analysis in that aspect was impossible, albeit very important. There was a lack of laboratory information for some of the patients during the early phase of the pandemic, especially cultures for bacterial infection and inflammatory biomarkers. Data on this were sparse and inconsistent among institutions due to a lack of clear procedures for handling microbiological specimens early in the pandemic. Future studies should evaluate the rationale of antibiotic treatment based on bacterial culture identification and identify the possibility of antibiotic resistance in COVID-19 patients treated with antibiotics.

## Conclusions

This study demonstrated that antibiotic prescribing in Malaysia during the early phase of the pandemic was low compared to other countries. In line with the guidelines for antibiotic prescribing for community-acquired pneumonia to cover for bacterial co-infection during viral pneumonia, the choice of antibiotics given for COVID-19 patients suspected of having bacterial co-infection was amoxicillin/clavulanic acid, and the choice of antibiotic for escalation was piperacillin/tazobactam. Males with a comorbidity who were more ill, given anti-viral and anti-malarial agents, admitted to the HDU/ICU, prescribed corticosteroids, and having derange blood parameters such as neutrophil, lymphocyte, and CRP, associated with a higher odds of receiving antibiotics.

## Data Availability

The datasets used and/or analysed with permission to support the finding of this study are available from Clinical Research Centre Hospital Sungai Buloh but restrictions apply to the availability of these data, which were used under permission for this study, and so are not publicly available. Data, however, are available from the authors upon reasonable request and with permission of the Infectious Diseases Unit or Clinical Research Centre Hospital Sungai Buloh.

## Abbreviations

ICU: Intensive Care Unit
HDU: High Dependency Unit
CRP: C-reactive protein
